# Xylene: An overview of its health hazards and preventive measures

**DOI:** 10.4103/0973-029X.64299

**Published:** 2010

**Authors:** Reena Kandyala, Sumanth Phani C Raghavendra, Saraswathi T Rajasekharan

**Affiliations:** *Department of Oral Pathology, Vishnu Dental College, Bhimavaram - 534 202, Andhra Pradesh, India*; 1 *Department of Endodontics, Vishnu Dental College, Bhimavaram - 534 202, Andhra Pradesh, India*

**Keywords:** Toxicity of xylene, xylene substitutes, xylene

## Abstract

Xylene is an aromatic hydrocarbon known for its wide usage in tissue processing, staining and cover slipping in the histology laboratory. The hazards of xylene are well documented, making it a potential occupational hazard for the histopathological technicians. As every other profession became cautious of the occupational hazards, the very speciality that identifies the illnesses became one of the last to become aware and remedy its own hazards. This review article aims to discuss the toxicity of xylene and safety measures to counteract the hazards and enlists the pros and cons of using various substitutes that claim to be much safer, better and faster.

## INTRODUCTION

Xylene is an aromatic hydrocarbon widely used in industry and medical technology as a solvent. It is a colorless, sweet-smelling liquid or gas occurring naturally in petroleum, coal and wood tar, and is so named because it is found in crude wood spirit (Gr. xy`lon- wood).[1] It has a chemical formula of C6 H4 (CH 3)2 and is referred to as “dimethyl benzene” because it consists of a six-carbon ring to which two methyl groups are bound. It exists in three isomeric forms: ortho-, meta- and para-xylene.[1]

Xylene is used as a solvent in the printing, rubber, paint and leather industries. It is found in small amounts in airplane fuel, gasoline and cigarette smoke. In dentistry, xylene is used in histological laboratories for tissue processing, staining and cover slipping and also in endodontic retreatment as a guttapercha solvent. Its high solvency factor allows maximum displacement of alcohol and renders the tissue transparent, enhancing paraffin infiltration. In staining procedures, its excellent dewaxing and clearing capabilities contribute to brilliantly stained slides.[1]

Laboratory-grade xylene is composed of m-xylene (40–65%), p-xylene (20%), o-xylene (20%) and ethyl benzene (6-20%) and traces of toluene, trimethyl benzene, phenol, thiophene, pyridine and hydrogen sulfide. Histopathological technicians who routinely come in contact with xylene-contaminated solvents in the workplace are the population most likely to be exposed to high levels of xylene. The current Occupational Safety and Health Administration permissible exposure limit for xylene is 100 ppm as an 8-h time-weighted average (TWA) concentration.[2] The National Institute for Occupational Safety and Health recommended exposure limits for xylene at 100 ppm as a TWA for up to a 10-h work shift and a 40-h work week and 200 ppm for 10 min as a short-term limit.[3]

Besides occupational exposure, the principal pathway of human contact is via soil contamination from leaking underground storage tanks containing petroleum products. Xylene can leak into the soil, surface water or ground water where it may remain for months or more before it breaks down into other chemicals. However, as it evaporates easily, most of it goes into the air and gets broken down by sunlight into other less-harmful chemicals. Most people begin to smell xylene in air at 0.08–3.7 ppm (parts per million) and begin to taste it in water at 0.53–1.8 ppm.[1]

## TOXICITY OF XYLENE

Exposure to xylene can occur via inhalation, ingestion, eye or skin contact. It is primarily metabolized in the liver by oxidation of a methyl group and conjugation with glycine to yield methyl hippuric acid, which is excreted in the urine. Smaller amounts are eliminated unchanged in the exhaled air. There is a low potential for accumulation.[4,5] Xylene causes health effects from both acute (<14 days) and also chronic (>365 days) exposure. The type and severity of health effects depends on several factors, including the amount of chemical you are exposed to and the length of time you are exposed for. Individuals also react differently to different levels of exposure.[1]

## NERVOUS SYSTEM

The main effect of inhaling xylene vapor is depression of the central nervous system, with symptoms such as headache, dizziness, nausea and vomiting. The effects listed below can begin to occur with exposure to air levels of about 100 ppm. They are reversible and become more noticeable and serious as the length of time of exposure increases[1] [Table 1].

**Table 1:** Effect of xylene on the nervous system

100–200 ppm	Nausea, headache
200–500 ppm	Feeling “high,” dizziness, weakness, irritability, vomiting, slowed reaction time
800–10,000 ppm	Giddiness, confusion, clumsiness, slurred speech, loss of balance, ringing in the ears
>10,000 ppm	Sleepiness, loss of consciousness, death

Effect of xylene on the central nervous system is attributed to the liposolubility of xylene in the neuronal membrane. It has been suggested that xylene disturbs the action of proteins essential to normal neuronal function either by disruption of the lipid environment in which the membrane proteins function or by direct interaction with the proteins in the membranes.[6] It has been suggested that a metabolic intermediate like methyl benzaldehyde could be responsible for the toxicity of xylene. Oxidation of xylene to these intermediates by microsomal enzyme systems may occur in the brain.[6] Changes in the levels of various neurotransmitters and lipid composition have been observed in several brain areas following acute- and intermediate-duration exposure to xylene. It is unclear whether these represent direct effects of xylene or are secondary changes resulting from nonspecific central nervous system depression.[7,8]

Long-term exposure may lead to headaches, irritability, depression, insomnia, agitation, extreme tiredness, tremors, impaired concentration and short-term memory. This condition is sometimes generally referred to as “organic solvent syndrome.” Unfortunately, there is very little information available that isolates xylene from other solvent exposures in the examination of these effects.[2]

## EYES, NOSE AND THROAT

Irritation of the nose and throat can occur at approximately 200 ppm after 3–5 min. Accidental splash in the eye may damage the surface of the eye, which will heal within a few days.[1]

## LUNGS

Exposure to xylene at levels of 200 ppm or greater can irritate the lungs, causing chest pain and shortness of breath. Extreme overexposure (*e.g.*, in a confined space) can result in pulmonary edema, a potentially life-threatening condition in which the lungs fill with fluid. However, there is no evidence that repeated, low-level exposure has any long-term effects on the lung.[1]

## LIVER AND KIDNEY

At very high levels of exposure, xylene can injure the liver and kidneys, but this is extremely unlikely to happen without noticeable effects on the nervous system. Generally, such damage is reversible.[1] Low-level occupational exposure does not affect the liver and the kidneys.[9]

## BLOOD

There is no evidence that exposure to xylene affects the blood cells in humans. Earlier reports of low red blood cell counts (anemia) may have been due to contamination of xylene with benzene.[1]

## GASTROINTESTINAL TRACT

Symptoms of nausea, vomiting and gastric discomfort were observed in workers exposed to xylene vapors (unspecified concentration), which were reversible.[10]

## MUSCULOSKELETAL SYSTEM

Workers exposed to xylenes (TWA 14 ppm) reported reduced grasping power and reduced muscle power in the extremities more frequently than the unexposed controls. This is due to the neurological effect rather than a direct effect on the muscles.[9]

## SKIN

Xylene, like other organic solvents, can dissolve the skin’s natural protective oils. Frequent or prolonged skin contact can cause irritation and dermatitis, dryness, flaking and cracking of the skin. Damaged skin may allow greater absorption of chemicals.[11,12] Xylene easily penetrates most ordinary clothing and can become trapped in ordinary gloves and boots. Xylene trapped in the clothing can cause burns and blistering.[1]

## CANCER

There is inadequate evidence for the carcinogenicity of xylene in humans.[1]

## REPRODUCTIVE SYSTEM

The available animal information is insufficient to connect xylene with any reproductive effects.[13,14] Xylene has produced fetotoxic effects like delayed ossification and behavioral effects in animals, in the absence of maternal toxicity. Xylene inhaled by a woman can reach a developing fetus and can contaminate her breast milk. It is recommended that pregnant and nursing women minimize their exposure to xylene, just as they should minimize their exposure to alcohol, tobacco and other drugs.[1]

## PREVENTIVE MEASURES

- Substitution
- Local exhaust ventilation
- Proper protective equipment

### Substitution

Substitution means finding a substance that can perform the same function and which may lessen the hazard. Care should be taken not to introduce any new hazards when selecting a substitute for a hazardous material. After the hazardous effects of xylene became indisputable in the 1970s, many potential substitutes became available, some with as many if not more hazards. In general, these substitutes fall into four classes and are marketed under various tradenames. The chemical components are one of the following:[15]

- Limonene reagents
- Aliphatic hydrocarbon mixtures
- Aromatic hydrocarbon mixtures
- Mineral oil mixtures

#### Limonene reagents

Mainly composed of d-limonene, which is a hydrocarbon. It is the major component of citrus peel oils. Limonene is prepared by steam distillation of orange peels.[16] It has a strong citrus smell, variously described as pleasant, overwhelming, disgusting and allergenic and cannot be made odorless [Table 2].

**Table 2:** Advantages and disadvantages of limonene reagents

Advantages	Disadvantages
Biodegradable, noncorrosive, nonflammable (combustible)	Expensive
Contains no benzene and no toluene	Claims to be less toxic but the hazards are not well documented
Low toxicity levels	Offensive odor
Minimal tissue shrinkage	Very oily
Soluble in alcohol and mounting media	Incompatible with some of the mounting media
Reasonably fast drying and leaves no residue	Cannot be distilled
Reduced fire risk	Samples take more time to dry thoroughly
Has a high vapor pressure, and thus does not evaporate fast.	Degreasing effect on skin
Hence, cover slipping multiple slides is easy	

#### Aliphatic hydrocarbons

The term aliphatic means that these hydrocarbons are arranged in the form of a “chain” instead of being arranged in a “ring” (aromatic). Because of their aliphatic structure, the substitutes generally need more time to exact the same effect on the tissue as does their aromatic counterpart.[17] Some have much lower flash points than others and thus the fire hazard varies considerably. Different brands are available that differ considerably in chemical and physical properties, and distillation routines for one brand cannot be used with another brand[17] [Table 3].

**Table 3:** Advantages and disadvantages of aliphatic hydrocarbon mixtures

Advantages	Disadvantages
Most of them are odorless	Classified as hazardous waste due to flammability
Can be recovered by distillation	Not easily biodegradable
Less expensive than limonene reagents	More expensive than xylene
Nongreasy	(some of them)
Less irritating to the skin than xylene and d-limonene-based clearants	Less tolerant of contamination than xylene
Less toxic because they are catalytically hydrogenated to destroy the double bonds	

Much of the information about the substitutes has been obtained through the internet, from the material safety data sheets supplied by the manufacturers as well as the feedback of the technicians using them. Many laboratories are using the above-said substitutes for paraffin tissue processing during clearing and staining[18] as well as for frozen sections[19] satisfactorily, but still retain xylene for cover slipping and cleaning the tissue processors.

#### Aromatic hydrocarbon mixtures

Some high-boiling aromatic hydrocarbon mixtures having lower volatility than xylenes have been manufactured. These are not so popular because they are just as toxic as xylene.[15]

#### Mineral oil mixtures

Mineral (paraffin) oil mixtures look promising in eliminating xylene from most of the procedures. Isopropanol alone or mixed with molten paraffin is a technically acceptable and cost-effective substitute for xylene for tissue processing. It has been demonstrated that the best clearing agents from the sectioning quality and diagnostic value point of view, with automated or manual protocols, are mixtures of 5:1 and 2:1 isopropanol and mineral oil, followed by undiluted mineral oil, all at 50°C, making them a safer and cheaper substitute than xylene. Use of a 1.7% dishwasher soap aqueous solution at 90°C to dewax before staining and oven drying the stained sections before cover slipping can eliminate xylene from the staining tasks. Tissue processors’ retorts and conduits can be dewaxed with a 2% solution of a strong glassware laboratory detergent.[20] These four methodologies can make the histology laboratory xylene-free. Disposal of mineral oil and its mixtures is easily accomplished by mixing with the used paraffin and incinerating the resulting solid.[21]

### Local exhaust ventilation

The workplace can be modified to reduce the inhalational hazards by installing local exhaust ventilation with a proper hood.[22]

**Figure 1:**
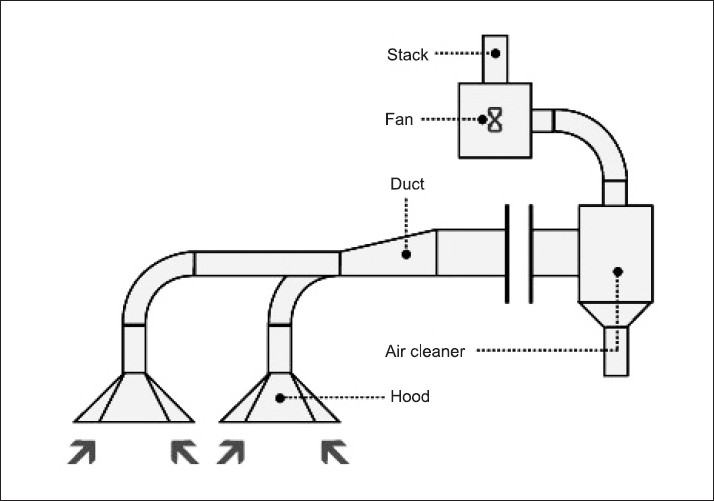
Local exhaust ventilation

Local exhaust ventilation is very effective in controlling the hazards because it removes the contaminant rather than diluting it. It should be in a fixed position, located close to the source of the hazard and have five key components [Figure 1]:

- A fan or a blower that provides enough negative air pressure to draw in contaminated air
- A hood that allows the effective capture of the contaminant
- A system of ducts that transport the contaminated air away from the workplace
- An air-cleaning device that removes the contaminants from the air
- A source of make-up air that replaces the air removed from the workplace

A well-designed hood takes advantage of the natural movement of the contaminant. As the air moves through the duct, it creates friction against the duct walls. Friction is greater at the corners, bends and obstructions of the duct. The overall duct length should be kept as short as possible with as few bends as possible. Various types of air-cleaning devices can be used, like fabric filters, charcoal filters, cyclones, electrostatic precipitators and scrubbers.[22]

### Proper protective equipment

Personal hygiene practices and protective equipment reduce the amount of a substance that is absorbed by the worker’s body after he or she has been exposed to it and also prevent hazardous toxic chemicals from being carried home. They include[2]

- thoroughly washing hands and removing outer protective clothing before entering clean areas
- usage of impervious clothing such as Buna–N-rubber or Viton gloves and impervious aprons
- a face mask or full-face organic respirator to reduce the inhalational hazards
- safety goggles/face shields for eye protection
- periodic medical examinations and biological monitoring of the worker’s body fluids to detect if the exposure to xylene is within limits.

## BIOLOGICAL MONITORING

Biological monitoring involves sampling and analyzing body tissues or fluids to provide an index of exposure to a toxic substance or metabolite. Xylene can be detected in the end-exhaled air, venous blood and the urine of exposed individuals. However, urinary levels of methylhippuric acid, a metabolite of xylene, appear to correlate better with airborne xylene concentrations than blood or breath concentrations of xylene.[23] Urinary concentrations of 1.5 g methyl-hippuric acid per gram creatinine in urine correlates with an 8-h exposure to an airborne concentration of 100 ppm xylene and a moderate level of work activity. Determination of a worker’s exposure to airborne xylene is made using a charcoal tube (100/50 mg sections, 20/40 mesh). Samples are collected at a maximum flow rate of 0.2 L/min until a maximum air volume of 12 L is collected. The sample is then treated with carbon disulfide to extract the xylene. Analysis is conducted by gas chromatography using a flame ionization detector. This method has a sampling and analytical error of 0.10.[1,2]

## EMERGENCY PROCEDURES

In the event of an emergency, remove the victim from further exposure, send for medical assistance and initiate the following emergency procedures:[1,24,25]

1. Eye exposure: If xylene or a solution containing xylene gets into the eyes, immediately flush the eyes with large amounts of water for a minimum of 15 min, lifting the lower and upper lids occasionally. Get medical attention as soon as possible
2. Skin exposure: The contaminated skin should be washed with soap and water for at least 15 min. If irritation persists, get medical attention
3. Inhalation: If xylene vapors are inhaled, move the victim at once to fresh air and get medical care as soon as possible. If the victim is not breathing, perform cardiopulmonary resuscitation; if breathing is difficult, give oxygen. Keep the victim warm and quiet until medical help arrives
4. Ingestion: If xylene or a solution containing xylene is ingested, give the victim several glasses of water to drink. Get medical help immediately. Keep the victim warm and quiet until medical help arrives. Do not induce vomiting if the person is unconscious as it is associated with the danger of pulmonary aspiration

## CONCLUSION

Efforts to reduce the health hazards in the histology laboratories should be made to create a safer working atmosphere by making the histopathology technicians more familiar with the health hazards of xylene, safety measures and emergency procedures. The hazards of xylene are well documented, but the substitutes are not so thoroughly evaluated. Most of the less-expensive alternatives to xylene do not have the same miscibility with alcohol, wax and resinous mountants, and nearly all are sold under trade names without any obvious disclosure of the chemicals of which they are composed. The assumption that they are safe just because the manufacturer says so is ill advised. It may not be comforting to get exposed on a daily basis to large volumes of a product of unknown chemical composition and largely untested health effects. Usage of proper personal protective equipment and a decent fume hood prevents the hazardous effects of xylene. In view of the established adverse effects of xylene, the Indian Association of Occupational Hygiene should make a law to safeguard the histopathology technicians against occupational hazards.
